# Alkali-Doped Nanopaper Membranes Applied as a Gate Dielectric in FETs and Logic Gates with an Enhanced Dynamic Response

**DOI:** 10.1021/acsami.2c20486

**Published:** 2023-02-03

**Authors:** Diana Gaspar, Jorge Martins, José Tiago Carvalho, Paul Grey, Rogério Simões, Elvira Fortunato, Rodrigo Martins, Luís Pereira

**Affiliations:** † AlmaScience Colab, Madan Parque, 2829-516Caparica, Portugal; ‡ CENIMAT/i3N, Department of Materials Science, NOVA School of Science and Technology, NOVA University Lisbon (FCT-NOVA) and CEMOP/UNINOVA, Campus de Caparica, Caparica2829-516, Portugal; § FibEnTech, Department of Chemistry, University of Beira Interior, 6201-001Covilhã, Portugal

**Keywords:** paper electronics, ionic doping, nanocellulose, cellulose-based FETs, cellulose-based logic gates

## Abstract

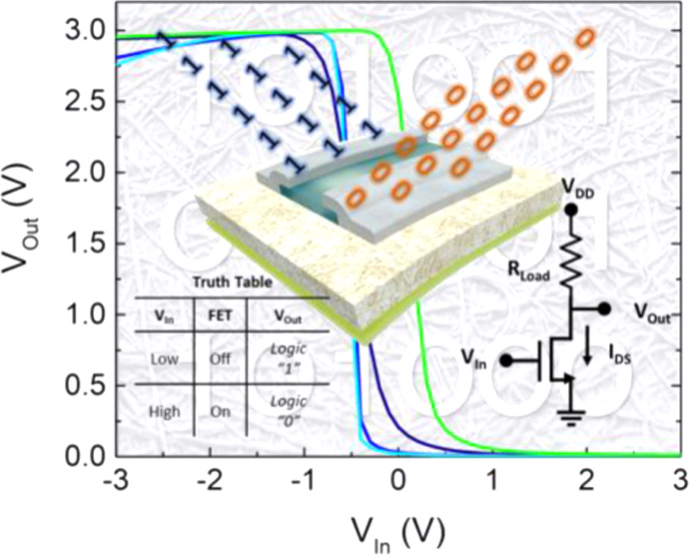

The market for flexible, hybrid, and printed electronic systems, which can appear in everything from sensors and wearables to displays and lighting, is still uncertain. What is clear is that these systems are appearing every day, enabling devices and systems that can, in the near future, be crumpled up and tucked in our pockets. Within this context, cellulose-based modified nanopapers were developed to serve both as a physical support and a gate dielectric layer in field-effect transistors (FETs) that are fully recyclable. It was found that the impregnation of those nanopapers with sodium (Na^+^) ions allows for low operating voltage FETs (<3 V), with mobility above 10 cm^2^ V^–1^ s^–1^, current modulation surpassing 10^5^, and an improved dynamic response. Thus, it was possible to implement those transistors into simple circuits such as inverters, reaching a clear discrimination between logic states. Besides the overall improvement in electrical performance, these devices have shown to be an interesting alternative for reliable, sustainable, and flexible electronics, maintaining proper operation even under stress conditions.

## Introduction

The interest in lightweight, flexible, and low-cost electronic devices has increased pioneering novel applications. Polyester-based polymers such as poly(ethylene naphthalate) (PEN) or poly(ethylene terephthalate) (PET) stand out as the most common substrates used in a large area and flexible electronics mainly due to their surface smoothness and compatibility with most processes used to make such devices and systems. Although the interesting properties offered by these petrol-based materials, recyclable and more sustainable alternatives are necessary for devices aimed to be low-cost and disposable.

Having in mind sustainability, circular economy, and end-of-life questions, the use of paper or other cellulose-based substrates as a platform for eco-friendly electronics became a reality in the last few years. The ubiquity of paper is unquestionable; this multifunctional material has been used for a long time and still is widely employed in a variety of applications in our daily life. Thus, thinking of it as part of “green” electronic devices in the future is a legitimate aspiration. Solid steps were taken during the last decade with the advent of a range of electronic^1−4^ or electrochemical^5−7^ devices manufactured directly onto paper substrates or from cellulose-based functional materials.^8,9^ Some examples are biosensors, energy storage, and conversion devices, or even crucial electronic building blocks such as diodes or field-effect transistors (FETs), demonstrating that the paper electronics era is establishing its foundations.^3,8,10−14^

Paper and other cellulose-based dielectrics can behave as a solid-state electrolyte that is ionically conducting and electronically insulating. If an external electric field is applied, the existing mobile ionic species can be slightly displaced and accumulated at the metal/paper interface (protons/cations), while at the opposite side, anions/electrons with equal densities are induced, creating cation/anion interfacial coupling electric double layers (EDLs).^15,16^ These EDLs present thicknesses of ≈1 nm and can be defined as nanometer-thick capacitors, reaching very high capacitances. Thus, when using paper membranes as the dielectric in FETs, the gate capacitance becomes insensible to the paper thickness.

In this work, modified nanopapers formed by modified micro/nanofibrillated cellulose were developed to be used as the gate dielectric in oxide-based FET with radically improved electrical performance. To achieve this, cellulose fibers were downsized using a top-down deconstruction which can be obtained using different types of mechanical processes, chemical or enzymatic pretreatments, or a combination of them.^17,18^ Such a cellulosic material has unique advantages over conventional paper made with larger fibers, namely, the smooth surface, flexibility, higher transparency, and dense packaging of the fibers that results in a matrix with lower porosity.

The use of the so-called nanopapers as the gate dielectric in transistors, either with organic or inorganic semiconductors, has already been reported in the literature.^19−21^ The novelty of the work reported in this manuscript work consists of the impregnation of the nanopaper with alkaline ionic species (lithium, sodium, and potassium), which results in an abrupt increase in the ionic conductivity and polarizability, which is determinant in the formation of EDL, dynamic response, and general performance of the transistors when used as a dielectric membrane and physical support of the same.

## Results and Discussion

### Characteristics of Alkali-Doped Nanopapers

**Figure 1:**
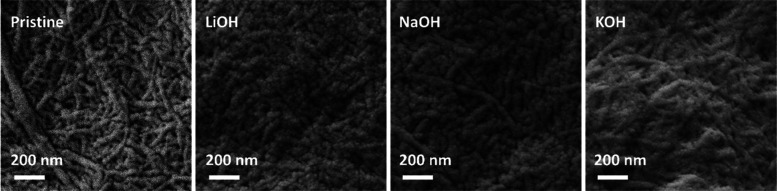
Pristine and alkali-doped NFC nanopapers produced with bleached birch kraft pulp.

The effect of protonation of the paper pulp before membrane formation when applying it as a gate dielectric in FETs was reported previously by our group.^22^ In this work, we explore dielectric membranes consisting of NFC (thickness ≈ 12 μm), or nanopaper, which possess a high internal surface area and produced to result in an RMS roughness in the range of 45–55 nm, optimized to be used as a gate dielectric on oxide-based FETs. In this work, we propose the impregnation of nanopapers with lithium (NFC:Li), sodium (NFC:Na), and potassium (NFC:K) ions with respective metal hydroxide solutions (0.5 M). The doping process changes the morphology of the membranes. The pristine papers present a matrix formed by small and entangled fibers (1–2 μm in length and up to 50 nm in width) that turns into a mat of swelled fibers without a defined shape (Figure 1), resulting from the nonregular swelling along the fibers (also denominated as a ballooning phenomenon) promoted by the alkali solutions.

**Figure 2:**
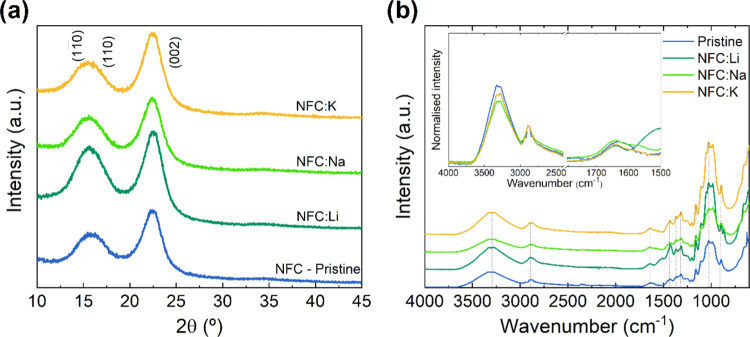
(a) XRD diffractograms of the pristine and ion-enriched nanopapers. (b) FTIR spectra of the pristine NFC and ion-doped NFC nanopapers.

Despite the morphology of the nanopapers being substantially modified with the addition of alkaline ions, the cellulose structure remains nearly unchanged. Figure 2a shows the XRD analysis of pristine and ionically doped nanopapers, revealing the characteristic peaks of cellulose I, (11̅0), (110), and (002), with no splitting of the last, while the crystallinity remains unaltered (≈60%). The molarity of the alkaline solution was optimized to improve the ionic conductivity of the nanopaper while avoiding any change in the structure of the cellulose fibers, that is, the conversion of cellulose I into cellulose II. Due to the small concentration of the alkali solutions used for the impregnation, no transformation in the cellulose structure was expected. It is reported that the onset for the transformation of cellulose I into cellulose II with NaOH concentrations is above 2.5 M, and for concentrations higher than 3.75 M of NaOH, the prevalent phase is cellulose II.^23,24^

The FTIR spectra of the pristine and impregnated nanopapers (Figure 2b) show the existence of characteristic bands of cellulose, such as absorption between 3000 and 3600 cm^–1^ assigned to the hydroxyl group −OH stretching, the C–H stretching at 2890 cm^–1^, the band 1430 cm^–1^ associated with H–C–H and O–C–H, and the band 1600–1635 cm^–1^ for the H–O–H bending vibration of the absorbed water molecules.^3,22,25,26^ The inset plot in Figure 2b shows the normalized FTIR spectra of all of the nanopapers. The data were normalized to the intensity of the C–H stretching band at 2890 cm^–1^, typically used as the baseline for the study of water content in the nanopapers. It could suggest that the ion-doped nanopapers have a reduced amount of the adsorbed water since the intensity of the 3000–3600 cm^–1^ band is slightly higher in pristine nanopapers. Moreover, the decrease in the intensity of this band can also indicate a decrease in the intra- or intermolecular hydrogen bonding since the contribution to this band also comes from the OH^–^ groups involved in the hydrogen bonds in cellulose. However, the ionic doping of nanopapers changes the way the absorption of water species occurs. The band at 1635 cm^–1^ is modified and the amount of absorbed water is indeed higher for NFC:Na, which suggests that the hydration shell of the alkali ions plays a role in water retention (and ultimately their mobility) in the nanopaper.

#### Electrochemical Characterization of the NFC Nanopapers

The high effective capacitance (*C*_eff_) achieved in these nanopaper membranes allows the operation of FET at a relatively low voltage when used as the gate dielectric. To assess *C*_eff_ and other electrochemical characteristics of the nanopaper membranes, electrolytic capacitors with the structure metal/insulator/metal (Al/nanopaper/Al) were produced and characterized by electrochemical impedance spectroscopy (EIS).

In the phase vs. frequency plot (Figure S1a), it is possible to identify three domains. For high frequencies, a dipole relaxation occurs as the phase angle (*Z*_phase_) < −45° and then a rise of *Z*_phase_ to values above −45° due to an ionic relaxation and a shift to a resistive response. At low frequencies, it shows a decrease of the *Z*_phase_, indicative of a capacitive response and EDL formation. This phenomenon leads to the increase of capacitance values at low frequencies. Therefore, to avoid the overestimation of parameters as the field-effect mobility and saturation mobility of the field-effect transistors, the capacitance used was determined at 10 mHz (Table S1).^27^

The *C*_eff_ in paper and other ionic materials is affected by several factors that can influence the formation of the EDL at the interface of the electrodes and dielectric. The displacement of ionic species toward those interfaces and consequent accumulation can depend on the water content, i.e., the capacitance of ionic dielectrics is significantly affected by water and ambient humidity levels,^22,27^ but also by the ions present in the matrix.^9^

The *C*_eff_ is greatly modified with ionic doping. The addition of salts results in the increase of *C*_eff_ from 0.98 ± 0.09 μF cm^–2^ for the pristine NFC and up to 2.77 ± 0.12 μF cm^–2^ for the NFC:Li (values listed in Table S1). In the Cole–Cole plots (Figure S1b), the semicircle diameter for high-frequencies decreases with the addition of ionic species, related to a reduction of the resistance from ≈2.4 MΩ cm^–1^ for the pristine membranes, down to ≈0.4 MΩ cm^–1^ for the nanopapers doped with Na^+^.

The acquired data were fitted with an equivalent circuit model (ECM) (Figure S1b). In these nanopapers, the high and intermediate frequency range can be modeled by a resistor (*R*_c_) related to the contact resistance between the nanopaper and the metal electrodes, in series with an RC circuit. This RC circuit is formed by a resistor (*R*_p_) in parallel with a constant phase element 1 (CPE1). The last is able to modulate the dipole and ionic relaxation, which takes place at the bulk of the nanopaper.^9,28^ Finally, a second constant phase element (CPE2) is needed to fit the data in the low-frequency domain, which is associated with EDL formation owing to the free charge accumulation at the metal/nanopaper interface. CPEs are used to modulate the nonideal capacitor behavior attributed to inhomogeneity, heterogeneous interface, surface roughness, and porosity of the nanopapers.^29^

### NFC-Gated Field-Effect Transistors

**Figure 3:**
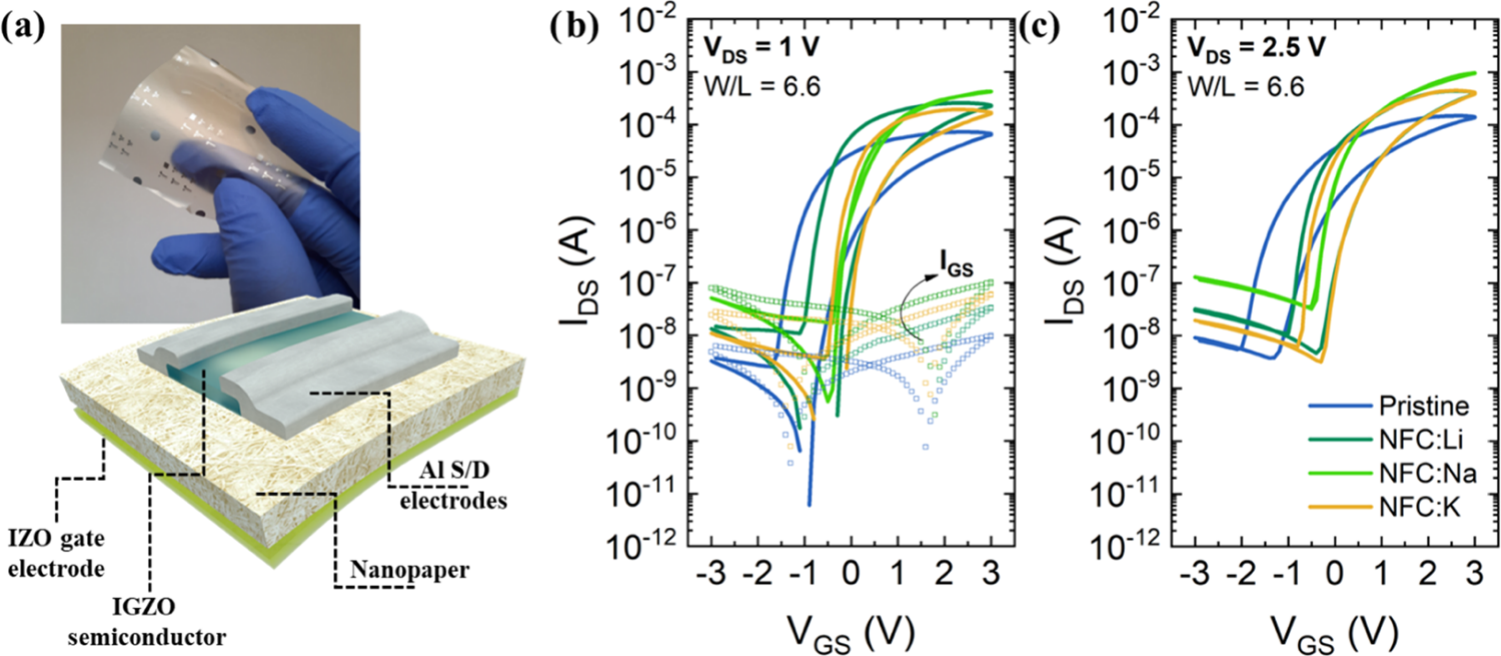
(a) Photo of NFC-gated FETs and schematic representation of the architecture. Transfer characteristics (*I*_DS_–*V*_GS_) with a hysteresis of the NFC-gated FETs for (b) *V*_DS_ = 1 V and (c) *V*_DS_ = 2.5 V.

The application of nanopapers as the gate dielectric was successfully demonstrated^3,21^ and it is undeniable that such membranes have better dielectric characteristics than the “conventional” paper.^1,22^ The modified NFC nanopapers developed in this work were used as the gate dielectric in IGZO FETs produced in a bottom staggered gate-like structure (channel width *W* = 1390 μm and length = 210 μm) (Figure 3a).

Figure 3b,c shows the *I*_DS_–*V*_GS_ transfer characteristics of the transistors for *V*_DS_ = 1 and 2.5 V and *V*_GS_ = [−3;3] V. The transistors produced on a pristine nanopaper present a current modulation of 4 orders of magnitude, a steep sub-threshold slope (*S*) of 0.29 ± 0.04 V dec^–1^, field-effect mobility (μ_FE_) of 4.32 ± 0.31 cm^2^ V^–1^ s^–1^, and a counter-clockwise hysteresis. The negative turn-on voltage (*V*_On_) of about −1.4 V denotes a depletion mode behavior (Figure 3 and Table 1), which was already reported for paper, cellulose nanocrystals (CNCs), and NFC-gated FETs.^2,22,30^

**Table 1:** Electrical Properties of NFC-Gated FETs for *V*_DS_ = 2.5 V

	Pristine	NFC:Li	NFC:Na	NFC:K
μ (cm^2^ V^–1^ s^–1^)	4.32 ± 0.31 (FE^a^)	4.98 ± 0.53	17.02 ± 1.08	13.20 ± 0.46
*V*_on_ (V)	–1.4 ± 0.2	–0.3 ± 0.1	–0.6 ± 0.1	–0.3 ± 0.2
Δ*V*_hysteresis_	1.2 ± 0.2	1 ± 0.1	0 ± 0.1	0.8 ± 0.1
*I*_On_/*I*_Off_	(3.6 ± 1.12) × 10^4^	(5.4 ± 0.26) × 10^4^	(2.7 ± 0.41) × 10^4^	(1.2 ± 0.37) × 10^5^
*S*(V dec^–1^)	0.29 ± 0.04	0.18 ± 0.07	0.17 ± 0.05	0.15 ± 0.05

a FE: field-effect mobility.

The impregnation with alkaline ions slightly shifts V_On_, but the depletion mode (normally on) remains. However, it results in the improvement of the devices’ performance. Despite the *I*_On_/*I*_Off_ is not significantly modified, the saturation mobilities (μ_sat_) for FETs on the NFC:Na nanopaper increases up to 17.02 ± 1.08 cm^2^ V^–1^ s^–1^, while the hysteresis (Δ*V*_hysteresis_) is almost eliminated. The devices on the NFC:K nanopaper dielectric reached 5 orders of magnitude *I*_On_/*I*_Off_ modulation with a considerable decrease of the sub-threshold slope down to 0.15 ± 0.05 V dec^–1^ (Figure 3c and Table 1) as a result of the increase in the *C*_eff_.

The transistors produced with NFC:Na present the highest off-current that is related to the electrical properties of the nanopaper. It was verified by EIS that this membrane has the highest conductivity resulting in a high charging current associated with a high capacitance that is responsible for the accentuated increase in the *I*_Off_ (Figure 3c). The trend observed for the FETs leakage current is validated by the resistivity calculated by EIS (see Table S1).

The hysteresis is counter-clockwise, associated with the accumulation of ionic charges in the semiconductor/nanopaper and nanopaper/gate electrode interfaces (Figure 3), with the lowest value obtained for FETs on NFC:Na membranes due to higher ionic conductivity.

Figure S2 shows the output curves (*V*_DS_–*I*_DS_) of the FETs. Despite the smoothness of the nanopaper, which is supposed to improve the contact between the S/D aluminum electrodes and IGZO, it is still possible to identify some current crowding for low *V*_DS_ values.

Considering the current state-of-the-art (Table S2), this work reports cellulose-gated FETs with good electrical performance, low operating voltages, and impressive electrical endurance and dynamic response (Figure S3). The devices were submitted to 900 consecutive cycles and still show proper current modulation (Figure S3), and parameters other than on-voltage such as *S* and μ_FE_ remain unchanged.

### Dynamic Response and Low-Voltage Logic on NFC Papers

#### Dynamic Response of NFC-Gated FETs

**Figure 4:**
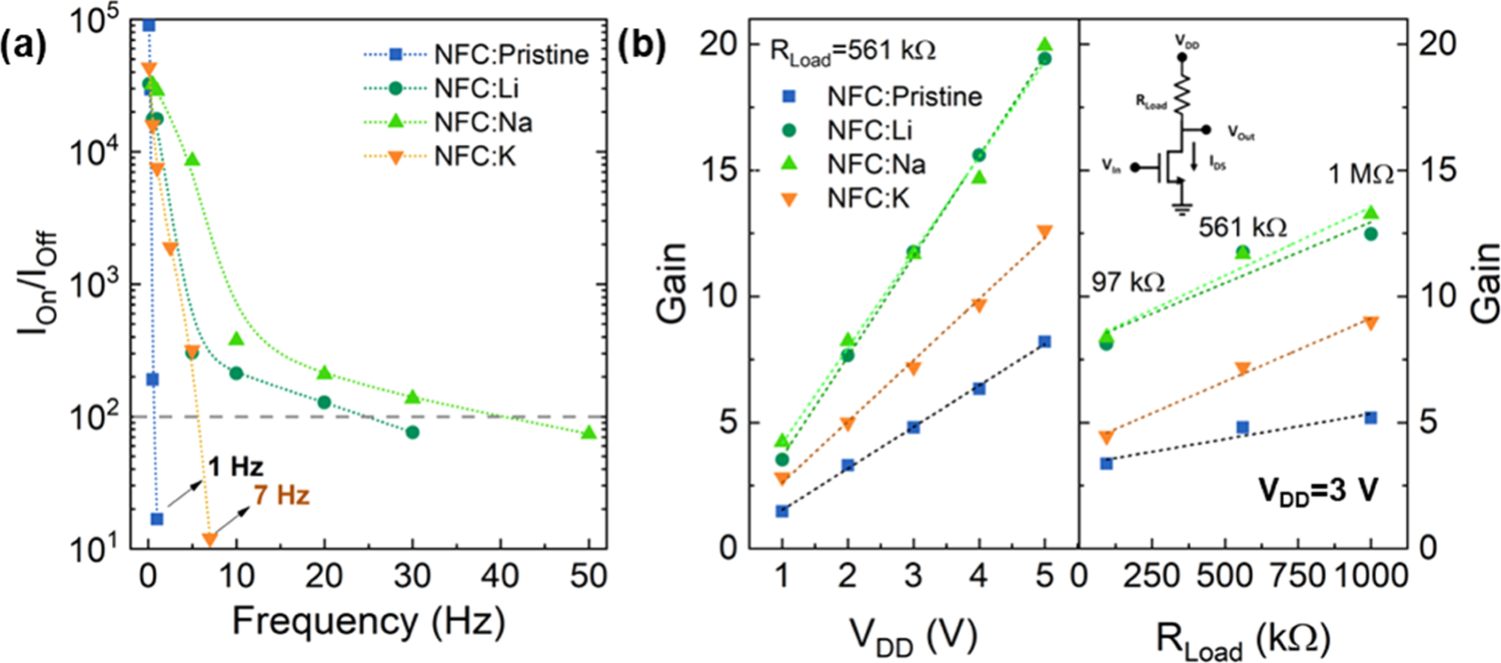
(a) *I*_On_/*I*_Off_ ratio as a function of the frequency applied to the gate of the NFC-membrane FETs (*V*_DS_ = 3 V). (b) Gain as a function of the applied *V*_DD_ = [1;5] V (on the left) and as a function of the load resistance used for constant *V*_DD_ = 3 V and −3 ≤ *V*_in_ ≤ 3 V (on the right). Inset: schematic diagram of the inverter.

As stated beforehand, the enhancement of the electrical performance of the FETs through the doping of the nanopapers with alkaline ions goes beyond the reduction of the hysteresis or increasing the mobility. The dynamic response of the NFC-gated FETs is substantially different according to the ions used. This response was evaluated by monitoring the *I*_On_/*I*_Off_ ratio while a square wave potential as gate voltage (*V*_GS_ = ±3 V for the pristine NFC, and [−2;1] V for the doped nanopapers) and a constant *V*_DS_ (*V*_DS_ = 3 V) were applied. Figure 4a shows the response of the devices produced on NFC:Pristine, where it is possible to properly discriminate a current modulation higher than 100 (up to 0.5 Hz), which is reduced to ≈10 at 1 Hz. The *I*_On_/*I*_Off_ ratio decay with the frequency allows us to conclude that the operation of the FETs produced with pristine membranes is restricted to 2–3 Hz according to the cutoff condition used.

The response of the FETs on ionically doped membranes is quite different (Figure 4a). The addition of ions has a tremendous impact on the operating frequency. The devices produced with those nanopapers show operation from 7 Hz for NFC:K up to around 50 Hz for NFC:Na when considering an *I*_On_/*I*_Off_ ratio of at least 2 orders of magnitude. Typically, the maximum operating frequency can be defined by the value where it is no longer possible to discriminate between the maximum and minimum current (*I*_On_/*I*_Off_ = 1).^22^ If considering so, the dynamic response of the FETs produced on NFC:Li and NFC:Na would be close to or even higher than 50 Hz (under the tested conditions: *V*_DS_ = 3 V and *V*_GS_ between −2 and 1 V). Extrapolating the *I*_On_/*I*_Off_ from Figure 4a, it is expected that the cutoff frequency for *I*_On_/*I*_Off_ = 1 would be around ≈40 and ≈66 Hz for the FETs on NFC:Li and NFC:Na, respectively (Figure S4).

#### Alkali-Doped NFC Papers in Inverters

The electrical reliability under a dynamic stimulus enables the use of these FETs in simple circuits. Inverters are one of the simplest yet fundamental logic gates which can be made by combining a single transistor with a resistor. In a different setup, the load resistor can be emulated using a load transistor, where both transistors can be either n-type (NMOS), p-type (PMOS), or a combination of n-type and p-type (CMOS).^3,31^

An inverter circuit composed of NFC-gated FETs combined with a load resistor was prepared, as schematically represented in the inset of Figure 4b. Ideally, the output voltage (*V*_Out_) should be equal to *V*_DD_ when the transistor is turned off, corresponding to a logic “1,” once this value is determined by *V*_Out_ = *V*_DD_ – *R*_Load_·*I*_DS_, while for logic “0,” the applied *V*_in_ increases the current level of the transistor. With a proper load resistor, the *R*_Load_·*I*_DS_ product should be the same as *V*_DD_ and subsequent reduction of the *V*_Out_ toward “0.”

The gain is defined by the shifting between logic “1” to logic “0” states and is determined as . The gain for the inverters produced on different membranes is plotted in Figure 4b, where it is possible to confirm the linear dependency on the *V*_DD_ applied. The inverters on the pristine nanopaper have the lowest gain (between ≈1.5 and 8). The ionic doping of the nanopaper results in a sharper transition between the logic states, corroborating the improvement of the dynamic response observed for the FETs. The steeper transition results in a higher gain that reaches 20 for the Li and Na-doped membranes (*V*_DD_ = 5 V). Besides the *V*_DD_, load resistance also plays a significant role in the gain of the inverters. Figure 4b shows the gain for inverters with FETs produced on different nanopapers as a function of the *R*_Load_ used (*V*_DD_ = 3 V). As expected, the gain follows the relation , increasing linearly with the load resistance (*R*_Load_ = 97 kΩ, 561 kΩ, and 1 MΩ).

**Figure 5:**
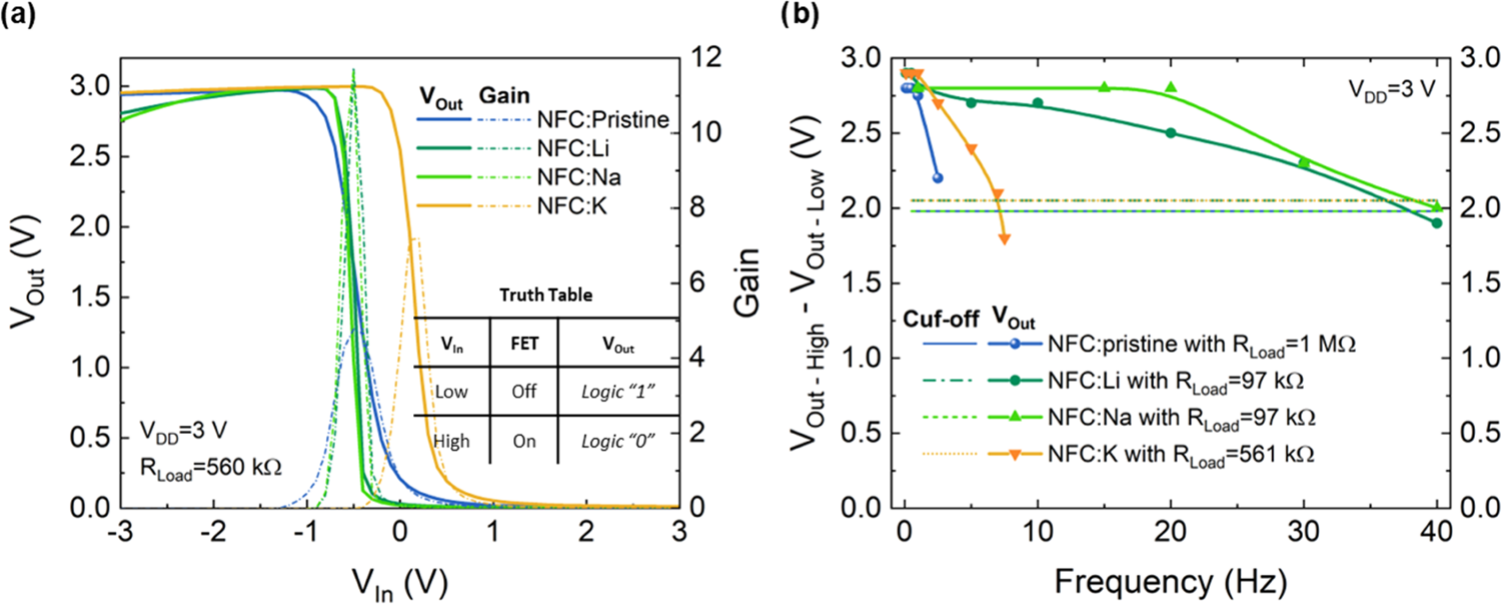
(a) Voltage transfer characteristics (VTCs) of the resistor-loaded inverters on NFC nanopapers measured with *V*_DD_ = 3 V. In the inset, the truth table for logic operation is shown. (b) Voltage output of the different NFC-based inverters with fixed *V*_DD_ = 3 V as a function of the frequency.

The voltage transfer characteristics (VTCs) in Figure 5a show a large discrimination between the two logic states, proving the excellent performance of the inverters for *V*_DD_ = 3 V and *R*_Load_ = 561 kΩ. For these parameters, the inverters made of FETs on the Li^+^ and Na^+^-doped nanopapers present a sharp transition between states and a gain of ≈12.

The values reported in this work are higher and differentiated from the ones found in the literature. For instance, CMOS devices produced on paper based on oxide semiconductors^31^ report a gain of about 16 but require *V*_DD_ = 17 V, while complementary inverters based on organic semiconductors and using NFCs^3^ as gate dielectric reach a gain of only 8.

For NFC:Li and NFC:Na nanopapers, the *V*_Out_ of the inverters is marginally lower than *V*_DD_, which is related to the higher *I*_DS_, compromising the proper turn-off of the devices, or to their increased *I*_GS_ (higher than for NFC:Pristine or NFC:K).

The dynamic response of the inverters is affected by the load resistance (Figure S4). The inverters were characterized using the same square wave as for the dynamic response of the FETs, *V*_In_ between −3 and 3 V for the pristine membrane, and *V*_In_ between −2 and 1 V for the ionically doped NFC nanopaper. The operation of NFC:Pristine and NFC:K is in line with the dynamic response of the FETs.

The pristine membranes allow for devices with a good distinction between logic states up to 1 Hz (*R*_Load_ = 1 MΩ), while above 1 Hz, the *V*_Out_High_ decreases once the device is not properly turned off, whereas the *V*_Out_Low_ starts to increase. The response of the inverters on NFC:K resembles the behavior of the pristine nanopapers. With low load resistances (*R*_Load_ = 97 kΩ), it is not possible to cut off the *I*_DS_ supplied by the FET and the logic “0” (*V*_Out_ = 0 V) is not reached. For higher load resistances, this behavior occurs for higher frequencies ensuring proper discrimination of high/low states up to 7.5 Hz (NFC:K). The best performance for the inverters is achieved for the NFC:Li and NFC:Na nanopapers (Figure S4) with excellent discrimination between logic “1” and logic “0” up to 40–50 Hz (Figure 5b).

The cutoff frequency was established at 70.7% (−3 dB) of the *V*_Out_High_ – *V*_Out_High_, determined for the lowest frequency and thus different for all membranes. According to the relation , different membranes present a threshold between 1.98 and 2.05 V. The dynamic performance of the devices produced with Li^+^- and Na^+^-doped nanopapers clearly shows superior performance and fast switching between states. In case of the need for higher amplification, either transistor resizing or a proper tuning to the load resistance should be considered.

### Second Life of Nanopapers

The impact of electronic waste is a concern and aspects such as the end of life, recyclability, or second life of electronic devices are nowadays a priority, where cellulose’s unique characteristics can be relevant. Having this premise in mind, some of the devices produced during this work on pristine NFC nanopapers were recycled to recover the NFC and produce new nanopaper membranes to be applied as the gate dielectric in transistors.

**Figure 6:**
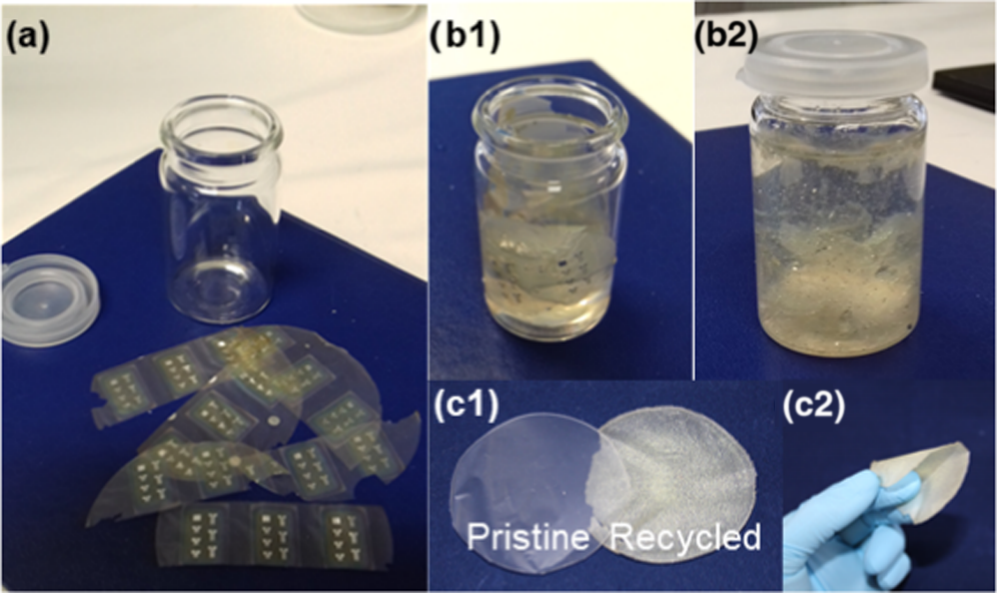
Steps involved in the recycling process of NFC FETs. (a) NFC-gated FETs used for recycling before being soaked in water (b1) and (b2) after stirring for a few hours. (c1) Dried pristine and recycled membranes, also photographed in (c2).

The process consisted of the dispersion and vigorous stirring of the nanopapers with the FETs in water to promote the separation of the nanofibers and disintegration of the inorganic thin films (Al, IZO, and IGZO). The suspension obtained was then poured into Petri dishes and left to dry at room temperature until total evaporation of the water and formation of the recycled nanopaper membranes. Figure 6 presents the different steps involved in the recycling/reusing process.

The new membranes have flexibility similar to the original pristine ones but are blurred (Figure 6c) due to the inclusion of Al and oxide particles coming from the thin films used to produce the FETs. The presence of such conductive particles can impact the performance of the new devices at various levels, namely, in the increase of the leakage current or the inclusion of particles with large dimensions (Figure S5) that could compromise the homogeneity of the thin-films deposited to produce the FET.

The capacitance of the recycled nanopaper calculated from EIS is ≈55 nF cm^–2^ at 0.01 Hz (Figure S6a). As previously mentioned, the use of paper as a dielectric relies on the transition from a resistive to a prevalent capacitive behavior. For the new nanopapers, the predominantly capacitive regime occurs for frequencies lower than 0.01 Hz, which is a limitation for application as the gate dielectric in FETs. Despite the inclusion of metal and oxide particles, no evident electrochemical reactions occur in the semiconductor/recycled-NFC interface. The CV measurements (performed with the same scan rate and range of voltages used for the FETs) (Figure S6b) did not show any evidence of redox-reaction peaks (associated with Faradaic currents).

The devices produced on the recycled nanopapers showed proper modulation (Figure S6c). However, the challenges previously pointed out have a great impact on the reproducibility and yield of these devices (produced with the same dimensions) also due to the inadequate quality of the S/D contacts. Regardless of the loss of performance of FETs produced on the recycled nanopaper when compared with pristine ones, it was possible to validate the recyclability/reusability of the nanofibers. The use of nanofibrillated cellulose and inorganic thin-films produced by physical vapor deposition (PVD) is challenging when trying to separate them due to the strong adhesion between the thin-films and the fibers. However, a recent work developed by our research group reported a successful separation and recycling process when using printed layers instead of PVD ones.^32^

## Conclusions

In this work, transistors using NFC nanopaper membranes as a gate dielectric were produced, resulting in highly stable FETs capable of maintaining their electrical performance under severe electrical endurance tests. The smooth and compact matrix of these nanopapers makes them an excellent substrate for flexible/bendable electronics. The addition of alkali metal ions to the cellulosic nanofibre matrix resulted in a notorious improvement in the electrical performance of the devices, with *I*_On_/*I*_Off_ ratios surpassing 5 orders of magnitude and saturation mobilities close to 17 cm^2 −1^ s^–1^, while the sub-threshold slope is decreased down to 0.15 V dec^–1^. The dynamic response of the devices is enlarged, where an *I*_On_/*I*_Off_ ratio of about 2 orders of magnitude is obtained up to ≈50 Hz for Na^+^-doped nanopapers. Thus, it was possible to evolve to the simplest logic circuit, an inverter, combining those FETs with the load resistance. For the Li^+^- and Na^+^-doped membranes, the inverters reached gains up to 20.

The use of NFC nanopaper membranes as the gate dielectric allowed for the reuse/recycling of FETs for the preparation of new nanopapers. The present work successfully supports the claims of sustainability foreseen for paper electronics. The process to reuse these nanopapers is not yet mastered, mostly due to the strong adhesion between the cellulose fibers and the PVD-deposited oxides and metal layer used to produce the FETs. However, it is expected that these preliminary results may open a new path toward eco-friendlier electronics.

## Experimental Section

### Fabrication of the Alkali-Doped Nanopapers

The undoped nanopaper was made using bleached birch kraft pulp with a solid content of 2%. The NFC gel was dispersed in deionized water and stirred for 30 min and then purred to a Petri dish and left to dry in a controlled environment (*T* = 25 °C with a relative humidity of ≈40%). After drying, the nanopapers were peeled off the dishes and ion impregnation was made using 10 mL of the lithium hydroxide solution (0.5 M), sodium hydroxide solution (0.5 M), and potassium hydroxide solution (0.5 M) as a source of the alkaline ions. The samples were kept in those solutions for 1 h and afterward rinsed in deionized water and left to dry.

### Alkali-Doped Nanopapers Characterization

The electrochemical characterization of the nanopapers was made using a Gamry Instruments Reference 600 potentiostat at room temperature. The electrochemical cells had a vertical configuration, where the cellulose was placed between two aluminum electrodes. EIS measurements were performed with 500 mV AC voltage in the frequency range 10 mHz to 1 MHz. CV measurements were made in the potential range between −3 and 3 V with a scan rate of 400 mV s^–1^

Fourier-transform infrared (FTIR) spectroscopy was performed using an attenuated total reflectance (ATR) sampling accessory (Smart iTR) equipped with a single-bounce diamond crystal on a Thermo Nicolet 6700 spectrometer. The spectra were acquired between 4000 and 650 cm^–1^ in steps of 4 cm^–1^. The crystallinity of the films was assessed by X-ray diffraction (XRD) using a PANalytical X′Pert PRO with Cu Kα radiation (λ = 1.540598 Å), while the morphology was investigated by scanning electron microscopy (SEM) with a ZEISS SEM/FIB AURIGA operated at 2 kV, with a working distance of 5.9 mm and an aperture size of 20 μm.

### Fabrication and Electrical Characterization of Devices on the Nanopaper

The devices were produced at room temperature in a staggered bottom gate FET structure. The nanopapers served as a gate dielectric, while the sputtered amorphous indium-gallium-zinc-oxide (30 nm of a-IGZO: In_2_O_3_–Ga_2_O_3_–ZnO; 2:1:2 mol %) was used as the semiconductor layer. The aluminum source and drain contacts (150 nm thick) were electron beam evaporated, while 200 nm indium-zinc-oxide (a-IZO) was sputtered to be used as the gate electrode. The devices were annealed in air at 150 °C for 60 min.

The FETs and inverters were electrically analyzed at room temperature in the dark using a microprobe station (Cascade Microtech M150) connected to a semiconductor parameter analyzer (Agilent 4155C). The dynamic electrical characterization at different frequencies (1–200 Hz) was done with the same equipment. The electrical parameters were extracted from the transfer characteristics. For the linear regime, the field-effect mobility (μ_FE_) is described as , where *W* is the channel width, *L* is the channel length, and *C*_i_ is the gate capacitance per unit area. For the saturation regime, the saturation mobility (μ_sat_) is described as . The sub-threshold slope can be determined from .

## Supporting Information Available

Electrochemical characterization of the NFC nanopapers; output characteristics of the NFC-gated FETs; state-of-the-art for the cellulose(based)-gated transistors; electrical endurance of the NFC-gated field-effect transistors; dynamic response: maximum operating frequency for the FETs on NFC:Li and NFC:Na; dynamic response of the inverters using alkali-doped NFC as the dielectric; nanopapers recyclability—SEM, EIS characterization, and FETs transfer characteristics (PDF)
