# Supplementary material for: Alkali-Doped Nanopaper Membranes Applied as a Gate Dielectric in FETs and Logic Gates with an Enhanced Dynamic Response
Source: ACS Appl Mater Interfaces. 2023 Feb 3;15(6):8319–26. doi: 10.1021/acsami.2c20486 (PMC9940104; doi:10.1021/acsami.2c20486)
Supplement: Supplementary file 1 — am2c20486_si_001.pdf [file am2c20486_si_001.pdf]

Supporting information

# Alkali-doped nanopaper membranes applied as gate dielectric in FETs and logic gates with enhanced dynamic response

*Diana Gaspar[1,2*], Jorge Martins[2], José Tiago Carvalho[2], Paul Grey[2], Rogério Simões[3], Elvira Fortunato[2], Rodrigo Martins[2], and Luís Pereira[*1,2].*

**Corresponding Authors**

***dgaspar@uninova.pt and lmnp@fct.unl.pt**

*Electrochemical characterisation of the NFC nanopapers*

The $C_{eff}$ was determined through the following equation [1]:

$$C_{eff} = -\frac{Z_{Imag}}{\omega \times |Z|^2} \quad (1)$$

Where $Z_{imag}$ is the imaginary part of the impedance, ω is the angular frequency, and |Z| is the absolute of the total impedance.

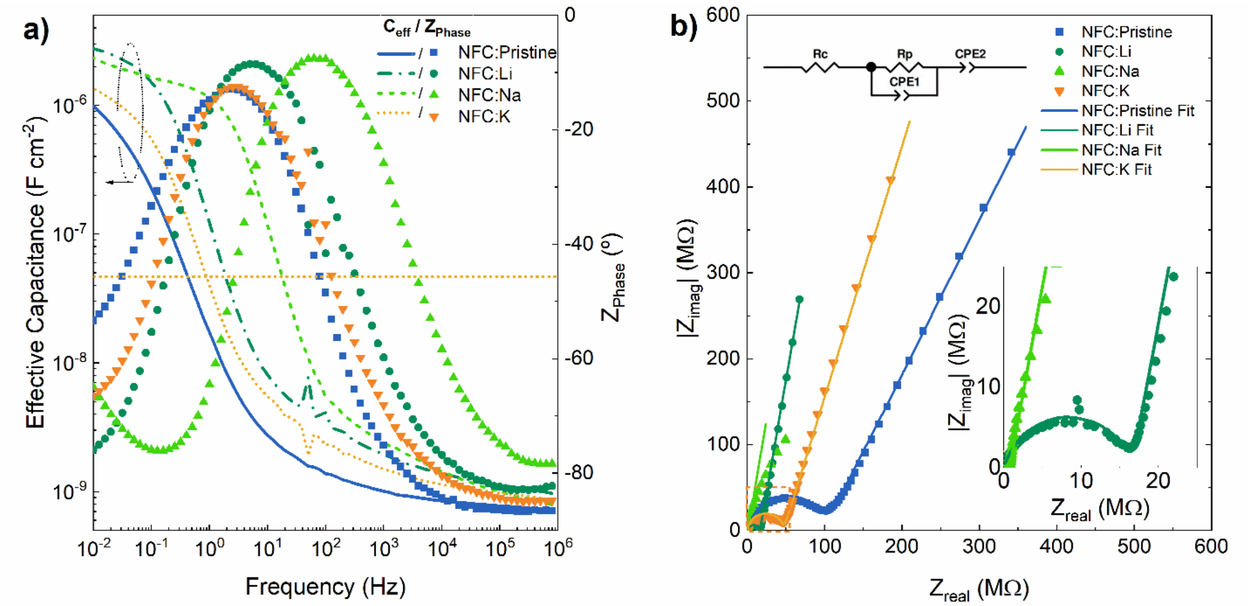


**Figure S1.** EIS of the NFC nanopapers (pristine, Li, Na and K doped): a) $C_{effl}$ (lines) and phase angle (open symbols) as a function of the applied frequency, and b) Cole-Cole plot of the NFC nanopapers and the equivalent circuit model (ECM). The inset shows a zoon of the indicated area for low resistance values from the NFC:Li and NFC:Na.

**Table S1** Summary of the electrochemical and electrical properties of the tested NFCs.

| | Pristine | NFC:Li | NFC:Na | NFC:K |
|---|---|---|---|---|
| Effective capacitance* (μF cm$^{-2}$) | 0.98 | 2.77 | 2.33 | 1.35 |
| *Resistivity (MΩ cm$^{-1}$)* | 2.37 | 1.19 | 0.4 | 0.9 |
| *Values determined for *f* = 10 mHz. | | | | |

*Output characteristics of the NFC-gated FETs*

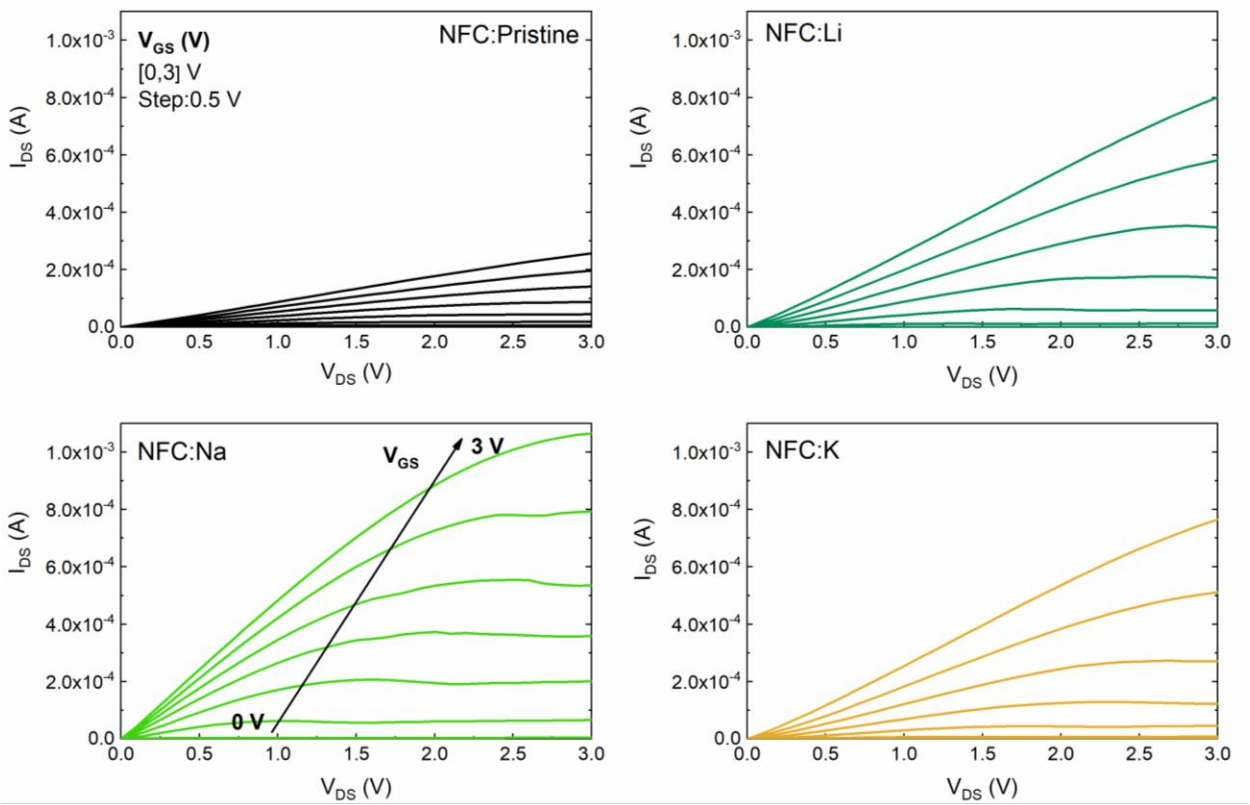


**Figure S2.** Output characteristics for the NFC-gated FETs.

*State-of-the-art for the cellulose(based)-gated transistors*

**Table S2** State-of-the-art for the cellulose(based)-gated transistors.

| Cellulosic substrate | Semiconductor S/D electrodes Gate | Deposition Techniques | Architecture | $V_{GS}$ range [V] | $V_{DS}$ [V] | $I_{ON}/I_{OFF}$ | $V_{On}$ / $V_{th}$ (V) | μ ($cm^2 V^{-1} s^{-1}$) | Cut-off frequency (Hz) | Year | Ref. |
|---|---|---|---|---|---|---|---|---|---|---|---|
| **Commercial paper** | IGZO \| Al \| IZO | Sputtering and e-beam | Staggered bottom gate | -20 to 20 | 15 | $2.9\times10^4$ | 1.9 ($V_{th}$) | 34 | N.A. | 2008 | 1 |
| **Paper with ionic resin matrix** | IGZO \| Al \| IZO | Sputtering and e-beam | Staggered bottom gate | -20 to 20 | 15 | $\sim10^4$ | 1 ($V_{On}$) | 40 | N.A. | 2008 | 2 |
| **Paper with ionic resin matrix** | IGZO \| Al \| IZO | Sputtering and e-beam | Staggered bottom gate | -10 to 20 | 15 | $3\times10^4$ | - | 29 | N.A. | 2009 | 3 |
| **Commercial paper** | IGZO \| Ti/Au \| IZO | Sputtering | Staggered bottom gate | -20 to 40 | 40 | $1\times10^4$ | 3.8 ($V_{th}$) | 35 | N.A. | 2009 | 4 |
| **Commercial paper** | $SnO_x$ \| Ni/Au \| IZO | Sputtering and e-beam | Staggered bottom gate | -20 to 20 | -30 | $1\times10^2$ | 1.4 ($V_{th}$) | 1.3 (holes mobility) | N.A. | 2013 | 5 |
| **Paper** | $In_2O_3$ \| $In_2O_3$ \| $In_2O_3$ | Pulsed electron beam | Staggered bottom gate | -5 to 5 | 5 | $6\times10^4$ | -3.6 ($V_{th}$) | 40000 | N.A. | 2013 | 6 |
| **Microfiber/Nanofiber cellulose paper** | IGZO \| Al \| IZO | Sputtering and e-beam | Staggered bottom gate | -15 to 15 | 15 | $7.5\times10^4$ | -4.8 ($V_{On}$) | 16 | >10 | 2014 | 7 |
| **Nanocrystalline cellulose** | IGZO \| Al \| IZO | Sputtering and e-beam | Staggered bottom gate | -30 to 30 | 15 | $2\times10^5$ | -8 ($V_{On}$) | > 7 | N.A. | 2014 | 8 |
| **Bacterial cellulose** | IGZO \| Al \| IZO | Sputtering and e-beam | Staggered bottom gate | -20 to 20 | 15 | $\sim10^4$ | - | - | N.A. | 2016 | 9 |
| **Office paper** | ZnO \| Ag \| Ag | Pen-writing and screen printing | Staggered bottom gate | -40 to 40 | 15 | $8\times10^1$ | 1 ($V_{On}$) | 4.45 | N.A. | 2017 | 10 |
| **Cellulose nanofibers electrolyte film as the gate dielectrics on paper substrate** | IZO \| IZO \| Ag | Sputtering | Planar dual gate | -0.8 to 1.5 | | $\sim10^7$ | 0.52 | 26 | 24 mHz | 2017 | 11 |
| **Tracing paper** | IGZO \| Al \| IZO | Sputtering and e-beam | Planar dual gate | -30 to 30 | 15 | $4\times10^4$ | < 0 ($V_{On}$) | 3 | 20 and 40 mHz (demonstrated in logic gates) | 2018 | 12 |

**Table S3 - Continuation**

| Cellulosic substrate | Semiconductor S/D electrodes Gate | Deposition Techniques | Architecture | $V_{GS}$ range [V] | $V_{DS}$ [V] | $I_{ON}/I_{OFF}$ | $V_{On}$ / $V_{th}$ (V) | μ ($cm^2\ V^{-1}\ s^{-1}$) | Cut-off frequency (Hz) | Year | Ref. |
|---|---|---|---|---|---|---|---|---|---|---|---|
| **Nanofibrillated cellulose (TEMPO-oxidized)** | C8 -BTBT \| Au \| Au | Thermal evaporation | Planar | 1.5 to -1.5 | -1 | ~$10^2$ | - | 1.25 (holes mobility) | 0.5 to 2.5 Hz | 2018 | [13] |
| **Nanofibrillated cellulose (TEMPO-oxidized)** | C8 -BTBT \| Au \| Au | Thermal evaporation | Planar (CMOS) | 3 to -10 | -5 | $3\times10^4$ | -1.26 ($V_{th}$) | 0.07 (holes mobility) | N.A. | 2018 | [14] |
| | NTCDI -F15 \| Au \| Au | | | -4 to 10 | 5 | $6.9\times10^3$ | 0.16 ($V_{th}$) | 0.01 | | | |
| **Crystalline nanocellulose** | CNT \| graphene \| graphene | Aerosol jet printing | Top gated | 2 to -2 | -0.5 | ~ $10^4$ | - | - | N.A | 2019 | [15] |
| **K-doped cellulose nanocrystals** | IGZO \| Al \| ITO | Sputtering and e-beam | Staggered bottom gate | -2 to 2 | 1.5 | ~ $10^6$ | < 0 ($V_{On}$) | 35 | N.A. | 2020 | [16] |
| **Li-doped cellulose acetate mats** | IGZO \| Al \| IZO | Sputtering and e-beam | Staggered bottom gate | -3 to 3 | 2 | $8.4\times10^2$ | -0.2 ($V_{On}$) | 22.8 | < 3Hz | 2021 | [17] |
| **Cellulose-nanofiber-soaked paper** | ITO \| ITO \| Ag | Sputtering and thermal evaporation | Planar dual gate | -1 to 2 | 2 | $7.5\times10^6$ | 0.32 ($V_{th}$) | 7.8 | 0.16 and 0.36 Hz (demonstrated in logic gates) | 2021 | [18] |
| **Cellulosic composite (mixture of fibres and cellulose-based ionic conductive hydrogel)** | ZnO \| C \| C | Screen printing | Planar | -0.5 to 2.5 | 1.2 | $3.3\times10^3$ | 0.37 ($V_{On}$) | 27.1 | 5-10 Hz | 2022 | [19] |
| **Nanofibrillated cellulose** | IGZO \| Al \| IZO | Sputtering and e-beam | Staggered bottom gate | -3 to 3 | 2.5 | Li:$5.4\times10^4$ Na:$2.7\times10^4$ K:$1.2\times10^5$ | ($V_{On}$) Li: -0.3 Na: -0.6 K: -0.3 | Li: 4.98 Na:17.02 K: 13.2 | Li: > 30 Na: > 50 K: ~7 | 2022 | This work |

**N.A. – Not accessed**

*Electrical endurance of the NFC-gated field-effect transistors*

Devices produced in pristine nanopaper membranes were used to perform the static measurements in the dark and at atmospheric pressure (23 ∘C and relative humidity of 40%). The devices were submitted to 900 cycles where the $V_{GS}$ was swept in the voltage range [-3;3]V with a step of 0.1 V, for $V_{DS}$=2.5 V. Then the devices were kept in the microprobe station to rest for 8 hours (in the dark), and further 200 cycles were applied. The influence of the scan rate was also checked by fixing it at 0.4 mV s$^{-1}$, and 4 V s$^{-1}$. After the 900 cycles, the devices still show proper current modulation, although the differences observed are highly related to the scan rate used. A successive decrease in $I_{Off}$ and $I_{GS}$ was monitored with the increase in the performed cycles (**Figure S3**). Parameters other than On voltage such as S and $\mu_{FE}$ remain nearly unchanged. After 8 hours of recovery, another $V_{GS}$ scan was applied, where $I_{Off}$ recovered to the initial value, being one order of magnitude lower than for the first curve (**Figure S3**).

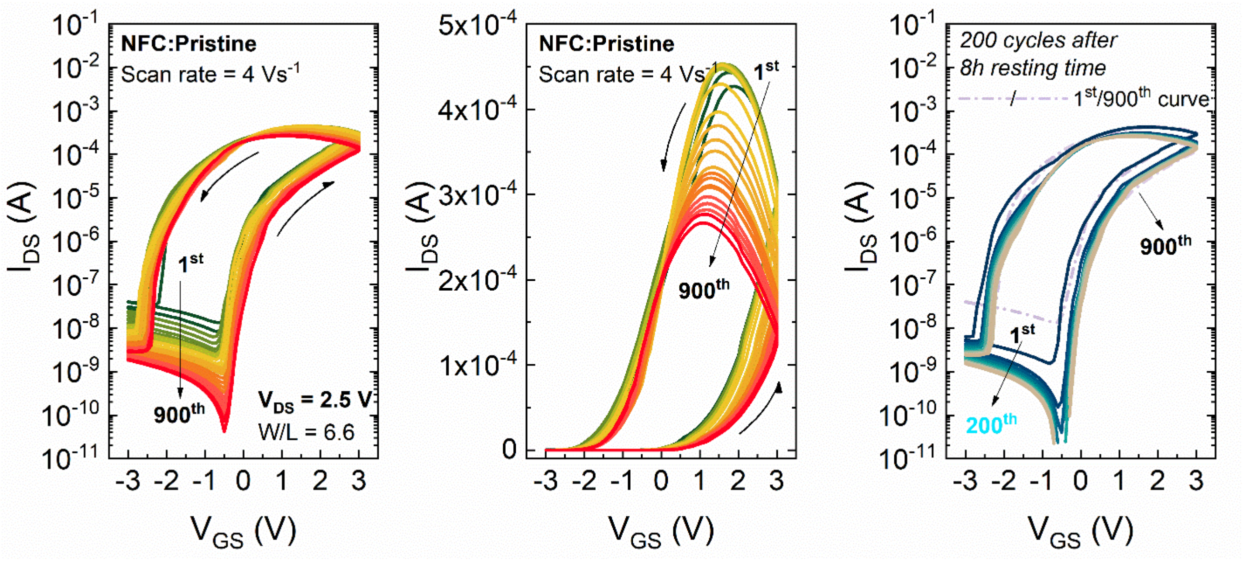


**Figure S3**. Transfer characteristic curves of the FETs characterised with a scan rate of 0.4 V s$^{-1}$. On the left, the curves for the initial 900 cycles are represented. On the right the 200 cycles after 8 hours of recovery. The dash-dot lines are indicative of the 1st and 900th cycle of the FET before resting time.

*Dynamic response: maximum operating frequency for the FETs on NFC:Li and NFC:Na*

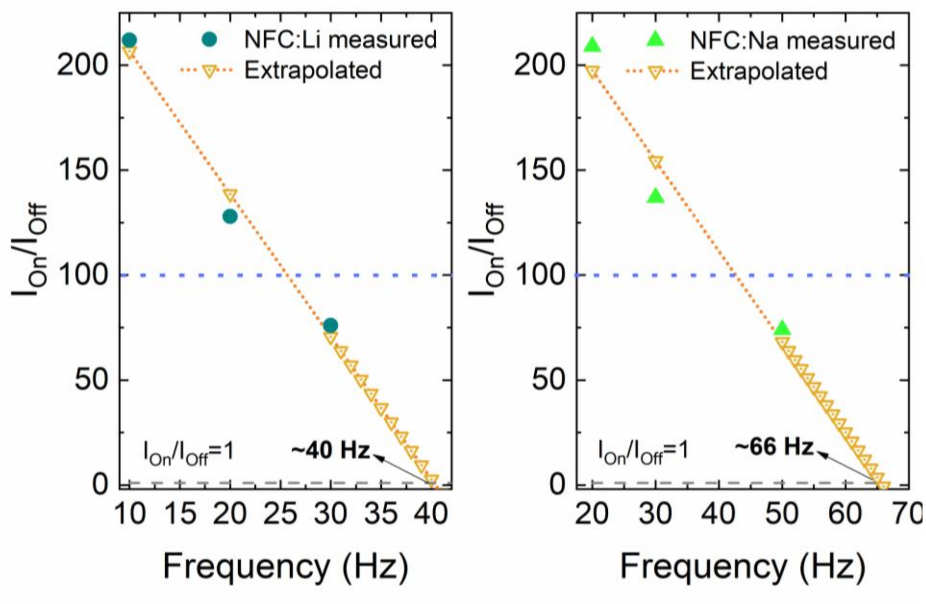


**Figure S4**. Extrapolation of maximum operating frequency for the FETs on Li-doped and Na-doped nanopapers.

*Dynamic response of the inverters using alkali-doped NFC as dielectric*

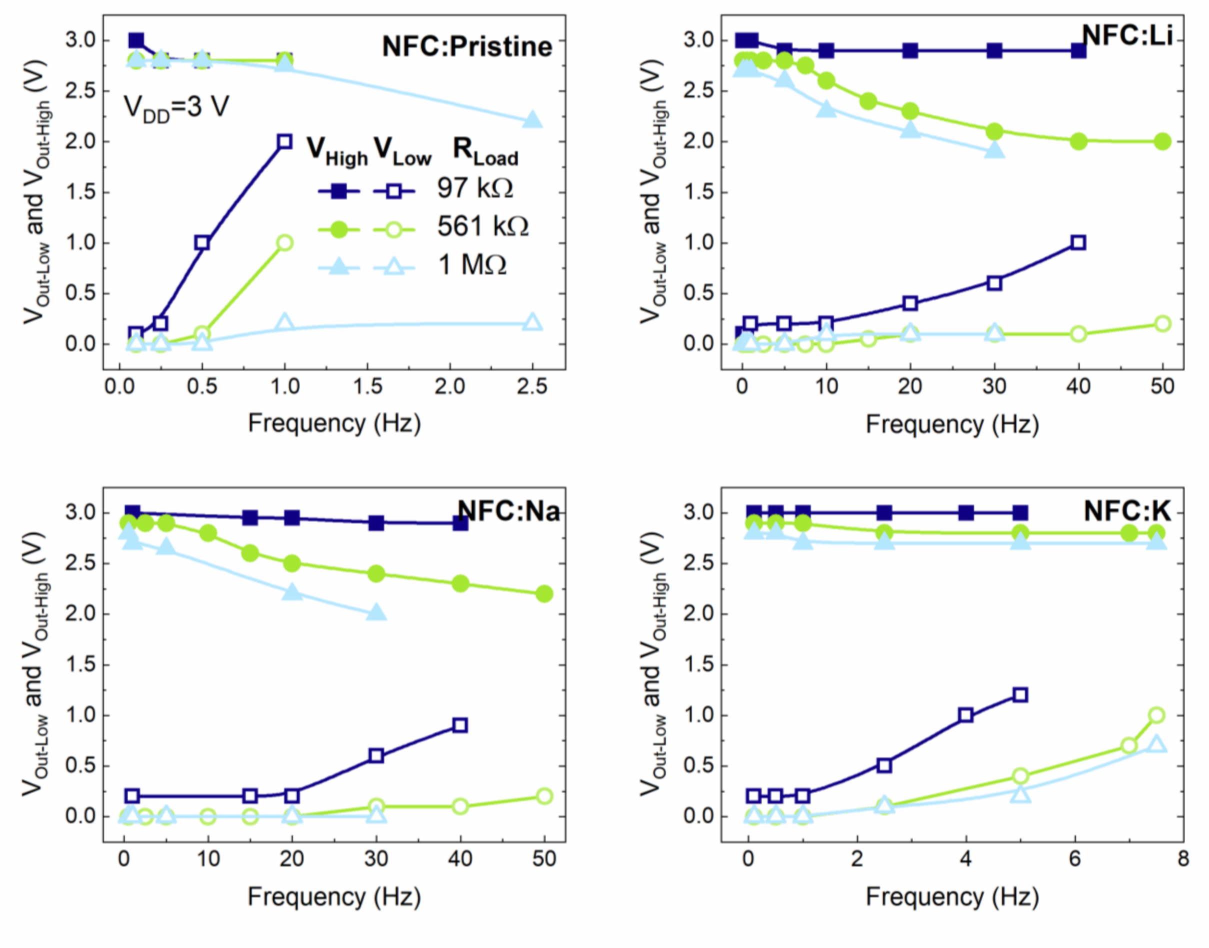


**Figure S5.** Voltage output for high and low states of the different NFC-based inverters with fixed $V_{DD}$=3 V and variable load resistances ($R_{Load}$ = 97 kΩ, 561 kΩ and 1 MΩ).

*Nanopapers recyclability*

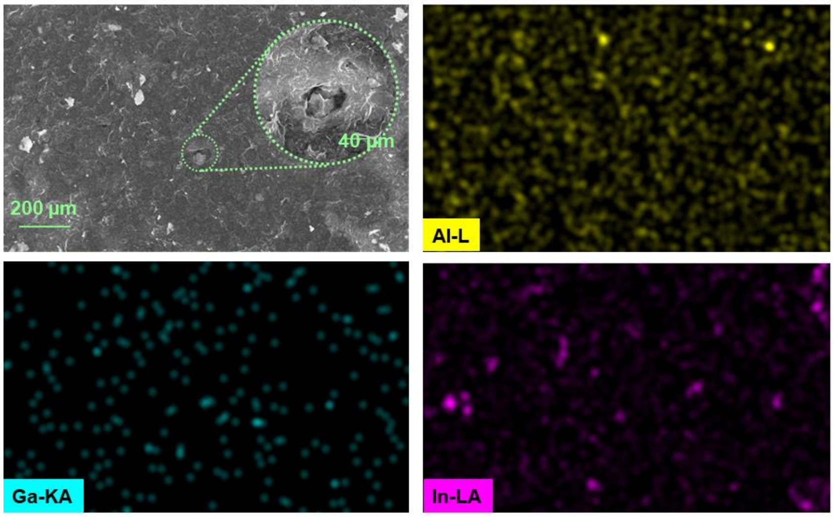


**Figure S6.** SEM micrograph of the recycled nanopapers, and EDX elements identification for the Al, In, and Ga. The inset shows an IZO particle with a large dimension entangled in the NFC fibres.

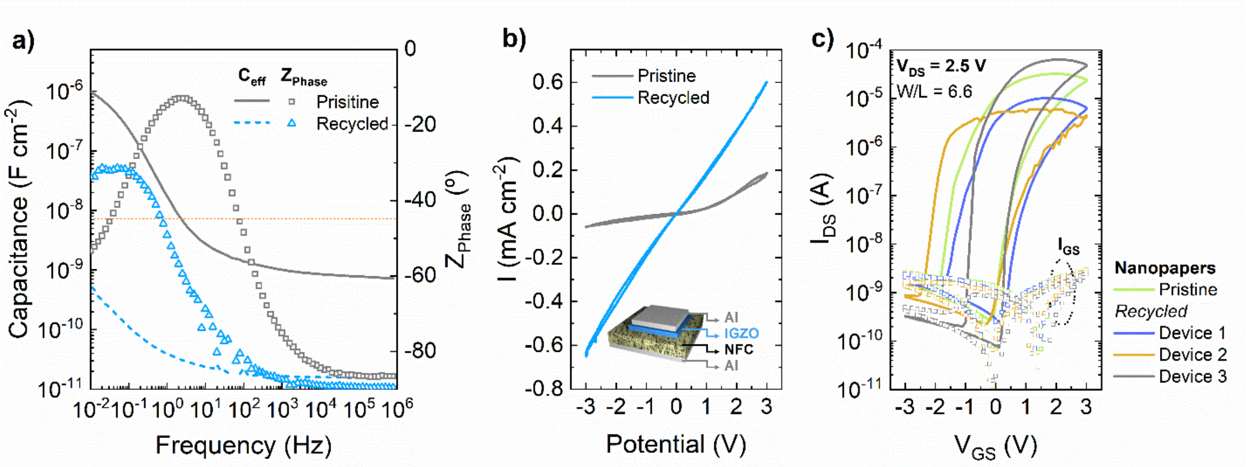


**Figure S7 a)** EIS characterisation of the NFC nanopapers (pristine and recycled), b) CV of the devices produced to emulate the electrochemical behaviour of real FETs (scan rate=0.4 mV $s^{-1}$) The inset shows a schematic representation of the produced cells. c) $I_{DS}$-$V_{GS}$ curves of the NFC-gated transistors using pristine and recycled membranes ($V_{GS}$=2.5 V).

## References


(1) Dasgupta, S.; Stoesser, G.; Schweikert, N.; Hahn, R.; Dehm, S.; Kruk, R.; Hahn, H. Printed and Electrochemically Gated, High-Mobility, Inorganic Oxide Nanoparticle FETs and Their Suitability for High-Frequency Applications. *Adv. Funct. Mater.* **2012**, *22* (23), 4909–4919.

(2) Fortunato, E.; Correia, N.; Barquinha, P.; Pereira, L.; Goncalves, G.; Martins, R. High-Performance Flexible Hybrid Field-Effect Transistors Based on Cellulose Fiber Paper. *IEEE Electron Device Lett.* **2008**, *29* (9), 988–990.

(3) Martins, R.; Barquinha, P.; Pereira, L.; Correia, N.; Gonçalves, G.; Ferreira, I.; Fortunato, E. Write-Erase and Read Paper Memory Transistor. *Appl. Phys. Lett.* **2008**, *93* (20), 203501.

(4) Martins, R.; Barquinha, P.; Pereira, L.; Correia, N.; Gonçalves, G.; Ferreira, I.; Fortunato, E. Selective Floating Gate Non-Volatile Paper Memory Transistor. *Phys. status solidi - Rapid Res. Lett.* **2009**, *310* (9), 308–310.

(5) Lim, W.; Douglas, E. a.; Kim, S.-H.; Norton, D. P.; Pearton, S. J.; Ren, F.; Shen, H.; Chang, W. H. High Mobility InGaZnO4 Thin-Film Transistors on Paper. *Appl. Phys. Lett.* **2009**, *94* (7), 072103.

(6) Martins, R. F. P.; Ahnood, A.; Correia, N.; Pereira, L. M. N. P.; Barros, R.; Barquinha, P. M. C. B.; Costa, R.; Ferreira, I. M. M.; Nathan, A.; Fortunato, E. E. M. C. Recyclable, Flexible, Low-Power Oxide Electronics. *Adv. Funct. Mater.* **2013**, *23* (17), 2153–2161.

(7) Gherendi, F.; Nistor, M.; Mandache, N. B. In2O3 Thin Film Paper Transistors. *IEEE/OSA J. Disp. Technol.* **2013**, *9* (9), 760–763.

(8) Pereira, L.; Gaspar, D.; Guerin, D.; Delattre, A.; Fortunato, E.; Martins, R. The Influence of Fibril Composition and Dimension on the Performance of Paper Gated Oxide Transistors. *Nanotechnology* **2014**, *25* (9), 094007.

(9) Gaspar, D.; Fernandes, S. N.; De Oliveira, A. G.; Fernandes, J. G.; Grey, P.; Pontes, R. V.; Pereira, L.; Martins, R.; Godinho, M. H.; Fortunato, E. Nanocrystalline Cellulose Applied Simultaneously as the Gate Dielectric and the Substrate in Flexible Field Effect Transistors. *Nanotechnology* **2014**, *25* (9).

(10) Fortunato, E.; Gaspar, D.; Duarte, P.; Pereira, L.; Águas, H.; Vicente, A.; Dourado, F.; Gama, M.; Martins, R. *Optoelectronic Devices from Bacterial NanoCellulose*; Elsevier B.V., 2016.

(11) Grey, P.; Gaspar, D.; Cunha, I.; Barras, R.; Carvalho, J. T.; Ribas, J. R.; Fortunato, E.; Martins, R.; Pereira, L. Handwritten Oxide Electronics on Paper. *Adv. Mater. Technol.* **2017**, *2* (6).

(12) Shao, F.; Feng, P.; Wan, C.; Wan, X.; Yang, Y.; Shi, Y.; Wan, Q. Multifunctional Logic Demonstrated in a Flexible Multigate Oxide-Based Electric-Double-Layer Transistor on Paper Substrate. *Adv. Electron. Mater.* **2017**, *3* (3), 1600509.

(13) Gaspar, D.; Martins, J.; Bahubalindruni, P.; Pereira, L.; Fortunato, E.; Martins, R. Planar Dual-Gate Paper/Oxide Field Effect Transistors as Universal Logic Gates. *Adv. Electron. Mater.* **2018**, *4* (12).

(14) Dai, S.; Wang, Y.; Zhang, J.; Zhao, Y.; Xiao, F.; Liu, D.; Wang, T.; Huang, J. Wood-Derived Nanopaper Dielectrics for Organic Synaptic Transistors. *ACS Appl. Mater. Interfaces* **2018**, *10* (46), 39983–39991.

(15) Dai, S.; Chu, Y.; Liu, D.; Cao, F.; Wu, X.; Zhou, J.; Zhou, B.; Chen, Y.; Huang, J. Intrinsically Ionic Conductive Cellulose Nanopapers Applied as All Solid Dielectrics for Low Voltage Organic Transistors. *Nat. Commun.* **2018**, *9* (1), 2737.

(16) Liu, Z.; Nie, S.; Luo, J.; Gao, Y.; Wang, X.; Wan, Q. Flexible Indium-Tin-Oxide

Homojunction Thin-Film Transistors with Two In-Plane Gates on Cellulose-Nanofiber-Soaked Papers. *Adv. Electron. Mater.* **2019**.

(17) Grey, P.; Fernandes, S. N.; Gaspar, D.; Deuermeier, J.; Martins, R.; Fortunato, E.; Godinho, M. H.; Pereira, L. Ionically Modified Cellulose Nanocrystal Self-Assembled Films with a Mesoporous Twisted Superstructure: Polarizability and Application in Ion-Gated Transistors. *ACS Appl. Electron. Mater.* **2020**, *2* (2), 426–436.

(18) Claro, P. I. C.; Cunha, I.; Paschoalin, R. T.; Gaspar, D.; Miranda, K.; Oliveira, O. N.; Martins, R.; Pereira, L.; Marconcini, J. M.; Fortunato, E.; Mattoso, L. H. C. Ionic Conductive Cellulose Mats by Solution Blow Spinning as Substrate and a Dielectric Interstrate Layer for Flexible Electronics. *ACS Appl. Mater. Interfaces* **2021**, *13* (22), 26237–26246.

(19) Williams, N. X.; Bullard, G.; Brooke, N.; Therien, M. J.; Franklin, A. D. Printable and Recyclable Carbon Electronics Using Crystalline Nanocellulose Dielectrics. *Nat. Electron.* **2021**, *4* (4), 261–268.

(20) Cunha, I.; Ferreira, S. H.; Martins, J.; Fortunato, E.; Gaspar, D.; Martins, R.; Pereira, L. Foldable and Recyclable Iontronic Cellulose Nanopaper for Low-Power Paper Electronics. *Adv. Sustain. Syst.* **2022**, *6* (9), 2200177.
